# Editorial

**Published:** 1853-04

**Authors:** 


### THE MEDICAL EXAMINER.

PHILADELPHIA, APRIL, 1853.

### DEATH OF PROFESSOR HORNER.

Dr. William E. Horner, Professor of Anatomy in the University of Pennsylvania, died at his residence in this city on the 13th of March last, in the 60th year of his age. His death, though somewhat sudden, was anticipated by his friends and family for some weeks; and, indeed, for many years, we believe, it had been apparent that organic disease was making slow but sure inroads on his naturally vigorous constitution.

No death, in the profession of this country, could have excited a more general sensation than that of Dr. Horner. Connected for thirty-three years with the University of Pennsylvania, as the adjunct and successor of Physick, he stood in the foremost rank among our teachers of national reputation ; while he was no less widely known as the author of a Treatise on Special Anatomy and Histology, as a constant contributor to our periodical medical literature, and as a skilful, original, and successful surgeon.

Dr. Horner was born on the 31st of June, 1793, at Warrenton, in Fauquier county, Virginia, where he received his early education. He afterwards spent some time in an Academy, at Dumfries, in Prince William County, in the same State, where he remained till his seventeenth year.

In 1810, he commenced the study of medicine with Dr. John Spence, of Dumfries, a Scotch physician and graduate of Edinburgh, who enjoyed considerable reputation as a practitioner.

In the autumn of 1812, he attended his first course of lectures in the University of Pennsylvania, and soon evinced his predilection for the study of anatomy, being occasionally employed in assisting the Demonstrator of Anatomy in the preparation of Prof. Wistar's lecture.

On the 3d of July, 1813, he obtained a commission as Surgeon's Mate in the Army of the United States; and was appointed to the regiment stationed at Fort Mifflin, near Philadelphia.

In the Spring of 1814, he graduated at the University, having presented an Inaugural Essay on Gunshot Wounds. He was ordered, in the Summer of this year, to the Niagara frontier, and was actively employed at Buffalo, Niagara, and Fort Erie. Our readers are familiar with his spirited and instructive reminiscences of this campaign, published in late numbers of the Examiner.

After the peace, in 1815, he was stationed at Norfolk, but he soon after resigned his commission, and returned to his native town, Warrenton, Virginia, where he entered upon the practice of his profession. It was not long, however, before he decided to seek a wider sphere, and, in November of the same year, he settled permanently in Philadelphia.

Dr. Horner's advancement here was almost without a precedent in rapidity. A stranger, without interest or influence, within two years from his establishment in Philadelphia, he entered the University as Demonstrator of Anatomy, under the most distinguished of American teachers; and three years afterwards, (in 1820,) he was appointed adjunct Professor of the same branch. In 1831, on the resignation of Dr. Physick, he was elected Professor of Anatomy in the University, and discharged the duties of this chair till within a short period of his death. From the date of his appointment to the adjunct Professorship, he devoted himself to the formation of an anatomical cabinet, which has gradually become one of the most splendid and complete in the world.

As a lecturer on anatomy, Dr. Horner was distinguished for clearness, perspicuity, and simplicity of style, a thorough familiarity with his subject, which secured entirely the confidence and attention of his hearers, and a manly, unaffected delivery. As a *teacher* he had no superior, (as we can bear grateful testimony.) To oratorical talent he made no pretensions, and had no claims.

Though up to a short period of his death, Dr. Horner continued in the steady discharge of his professional and other duties, he had for years suffered from dyspnœa, palpitation, and other symptoms, which left little doubt that he labored under cardiac disorder; and the emaciation of his frame made it evident that it was producing serious derangement of the functions of nutrition. For some weeks preceding his death, dropsical effusion had appeared. And, though within a day or two of his death, he was able to participate in the examination of students for degrees, yet we believe that neither himself nor his family entertained any hope that his life could be long protracted. The immediate termination, was, however, somewhat unexpected.

The *post-mortem* examination confirmed the diagnosis of disease of the heart. It was found very considerably hypertrophied and enlarged, being five and a half inches from the apex to the origin of the pulmonary artery, five and three-quarter inches in diameter, and thirteen and a half inches in circumference at its base. The tricuspid and mitral valves were healthy. The arch of the aorta was dilated and thickly ossified. There was also recent peritonitis, with streaks of fresh coagulable lymph over the peritoneal surface of the intestines. Perforation of the stomach or bowels had been suspected, as the cause of the peritonitis, but no traces of this lesion were found.

Dr. Horner's death, at this comparatively early age, is a loss which the profession of our country feel severely. As an anatomist and surgeon, the worthy successor of an illustrious predecessor, his place must long be vacant. But if not full of years, he went full of honors, leaving an unstained reputation in every personal and professional relation. It is gratifying too, to know, that his labors were not without that rare professional recompense—an ample fortune, which he owed exclusively to his own exertions. No man could have closed life under more consolations; and that greatest and best of consolations, a firm Christian hope, had been long and well secured.

---

St. Joseph's Hospital, *March* 17*th*, 1853.

At a Special Meeting of the Medical Board of St. Joseph's Hospital, held this day, for the purpose of taking action in regard to the death of their late President, William E. Horner, M. D., who departed this life on the 11th inst, it was unanimously

*Resolved*, That in Dr. Horner this Board have to lament one of the founders of the Hospital, a zealous and efficient advocate of its interests, and one of its most liberal benefactors, who spared neither his means, his labor, nor his skill, in furthering its welfare and in healing the diseases of its inmates; that in him they also mourn a colleague and a friend, who in all his intercourse was urbane and considerate, and ever prompt to sustain them by his influence and assist them by his counsel; one with whom it was a pleasure to associate and from whose exemplary candor they could always look for a just appreciation of their own acts.

*Resolved*, That a copy of this Resolution be furnished for publication in the Medical Examiner and the American Journal of the Medical Sciences. Alfred Stillé, Chairman.

J. Henry Smaltz, Secretary.

---

THE LATE DR. DANIEL DRAKE.

From a very interesting and eloquent Discourse on the Life, Character, and Services of Dr. Daniel Drake, by Prof. Gross, published in the February number of the *Western Journal of Medicine and Surgery*, we take the following particulars.

Dr. Drake was born at Plainfield, Essex County, New Jersey, on the

20th October, 1785. Two years and a half afterwards, his father emigrated to Kentucky, and settled at Mays Lick, twelve miles south-west of Maysville. The educational advantages of young Drake, during his boyhood, were very limited. In the autumn of 1800, at the close of his fifteenth year, he was sent to Cincinnati, and entered the office of Dr. Goforth, of that city, as a private pupil. He remained with this gentleman for four years, when he received from him an autograph diploma, under the sanction of which he practiced medicine for the next eleven years, "when it was corroborated by another from the University of Pennsylvania." In the autumn of 1805, he attended a course of lectures at the latter Institution, under Rush, Wistar, Barton, Physick, and Woodhouse. On his return, he practised medicine a year in Mason County, Kentucky, but soon afterwards went to Cincinnati. In 1807, he married; and after some years of professional occupation in Cincinnati, he, in 1815, attended a second course of lectures at the University of Pennsylvania, and graduated at the end of the session.

Dr. Drake's long and varied career, as a teacher of medicine, now commenced. A year after receiving his degree, he was appointed Professor of Materia Medica in the Medical Department of Transylvania University at Lexington. In 1819, he founded the Medical College of Ohio, at Cincinnati, he himself taking the Chair of Medicine. Misunderstandings with colleagues, however, resulted in Dr. Drake's retirement from this School; and in 1823, he resumed his old chair at Transylvania, passing two years later to the Chair of Medicine.

In 1827, Dr. Drake returned to Cincinnati and private practice. But, in 1830, be was called to the Professorship of Medicine in the Jefferson Medical College of Philadephia, where, according to Dr. Gross, he soon became the most popular Professor in the Institution, which then numbered both George McClellan and Eberle in its faculty. "Had Dr. Drake remained in the Jefferson School, no one can doubt," Dr. Gross observes, "that the brilliant success which has since awaited it would have been attained years before." But the restless energy of Dr. Drake's character impelled him to new schemes, and he returned to the West; and, after two unsuccessful attempts at medical organizations, in 1835, we find him at the head of a new and able Institution, with brilliant prospects—the Cincinnati Medical College. Dr. Drake, it appears, had greatly at heart the success of this enterprise. But after a brave struggle of four years, with classes of encouraging numbers, this organization was obliged to "succumb to the want of endowment."

Shortly after, he received an invitation to a chair, created with especial reference to him, in the University of Louisville, viz.: that of

Clinical Medicine and Pathological Anatomy. He filled this chair till 1844, when he was transferred to that of Medicine. In 1849, he withdrew from Louisville, and returned to Cincinnati to fill the chair of Medicine in the Medical College of Ohio. But "troubles either real or imaginary" brought Dr. Drake's career to a close here, after a single session. In 1850 we find him again at Louisville, and, in 1852, he finally left it, "once more to re-enter the Medical College of Ohio, now re-organized with an abler Faculty, and under brighter auspices." But the chequered career was now to be closed; and, "just at the opening of the session, full of hope and expectation about the classes and prospects of the Institution, the hand of death was laid upon him."

Dr. Drake was distinguished as a philanthropist, and was instrumental in founding many of the valuable benevolent institutions of the West. He was also foremost in promoting schemes of public improvement, and took a decided and active part in several of the great political questions of the day. He was emphatically, one of the *public men* of the West; a marked and influential citizen.

As a writer, he was voluminous, original, and industrious. He was frequently connected editorially with the medical journals of the West, and a steady contributor to them during his whole medical career. "But the most splendid exhibition of his genius is his work on the Diseases of the Interior Valley of North America, an enduring monument of his industry, his research, and his ability." As early as 1822, he issued a circular, requesting the co-operation of his professional brethren of the South-west, in furnishing material for this work.

"In 1837, fifteen years after the publication of his circular, he found, for the first time, sufficient leisure to enter vigorously upon the collection of materials for his long contemplated work In the summer of this year, accompanied by his two daughters, he visited a portion of the South for that purpose, during a tour of about three months. In 1843, he made a second tour, embracing Louisiana, Florida, Mississippi, Alabama, and the Gulf of Mexico; and subsequently he explored the interior of Kentucky, Tennessee, the two Carolinas, Virginia, Western Pennsylvania, New York, Illinois, Indiana, Michigan, Iowa, Wisconsin, Missouri, the great Lakes and Canada. Wherever he went his fame preceded him, and he was kindly received by his professional brethren, many of whom vied with each other to show him attention and hospitality. It was during his absence upon these missions, which he performed with the zeal of an apostle of science, that he wrote those numerous and interesting travelling editorials, as he styled them, for the Western Journal of Medicine and Surgery. These epistles, which form so conspicuous a feature of that periodical during the time referred to, were usually descriptive of the manners, habits, and diseases of the people among whom he wandered, of the climate, scenery, and productions of the country, and, in short, of whatever seemed, at the moment, to strike his fancy, or interest his mind.

"The materials thus collected were gradually digested and arranged, and finally presented to the profession, in the summer of 1850, under the elaborate title of "A Systematic Treatise, Historical, Etiological, and Practical, on the Principal Diseases of the Interior Valley of North America, as they appear in the Caucasian, African, Indian, and Esquimaux Varieties of its Population." The work, the first volume of which only has yet appeared, is illustrated by numerous charts and maps, designed and engraved at great expense, and was printed and published at Cincinnati under the author's immediate supervision. A second volume, the composition of which was nearly completed at the time of his decease, will be issued during the ensuing summer, under the care of a competent editor, and will be entirely devoted to subjects on practical medicine. The two together will constitute a monument of the genius and industry of their author, as durable as the mountains and the valleys, whose medical history they are designed to portray and illustrate. The toil and labor expended upon their production afford a happy exemplification of what may be accomplished by the well directed and persistent efforts of a single individual, unaided by wealth, and unsupported by the patronage of his own profession.

"It was originally the intention of Dr. Drake to comprise his work in two volumes; but as he progressed with the elaboration of his materials, he found that he should have a sufficiency for another; which now, that he is dead, will, of course, never be completed. The treasure remains, but the key wherewith to unlock it is gone.

"Every where at home the work received the most favorable commendation. All concurred in pronouncing it a performance of stupendous labor, and not a few of the leading journals declared that it was the most original production that our country had yet furnished. One of the most beautiful and appreciative notices of it appeared, soon after its publication, in the Daily Louisville Journal, from the pen of my colleague, Professor Yandell. Dr. Stillé, Chairman of the committee on Medical Literature, alluded to it in terms of high commendation in his report to the American Medical Association, at their meeting at Cincinnati, in May, 1850. Abroad, its appearance was hailed with similar feelings. The British and Foreign Medico-Chirurgical Review, one of the ablest and most discriminating periodicals of the day, published a long and highly flattering notice of it."

Dr. Gross pays the following beautiful tribute to the private virtues of his distinguished associate—admitting at the same time, however, that his ardent and positive temperament had made him enemies as well as warm, staunch, and admiring friends.

"His conduct, in all the relations of life, was most exemplary. In his friendships, usually formed with much caution, he was devoted, firm, and reliable, as many who survive him can testify. His attachments were strong and enduring. Few men, as he himself declared to me only a few months before his death, possessed so many ardent and faithful friends. His social qualities were remarkable. He loved his friends, enjoyed their society, and took great pleasure in joining them at the domestic board; where, forgetting the author and the teacher, he laid aside his 'sterner nature,' and appeared in his true character, plain and simple as a child, cheerful, amiable, and entertaining. It was during such moments, which served to relax the cords of his mind, and fit it for the renewal of its labors, that he shone to most advantage. His conversational powers on such occasions, as well as in the drawing-room, although superior, were not equal. Like all great and busy men, he had his cares and annoyance, his hours of depression and despondency, his fits of absence and restlessness."

